# Variability of Muscle Synergies in Hand Grasps: Analysis of Intra- and Inter-Session Data

**DOI:** 10.3390/s20154297

**Published:** 2020-08-01

**Authors:** Una Pale, Manfredo Atzori, Henning Müller, Alessandro Scano

**Affiliations:** 1 Swiss Federal Institute of Technology (EPFL), 1015 Lausanne, Switzerland; una.pale@epfl.ch; 2 Information Systems Institute, University of Applied Sciences Western Switzerland (HES-SO), 3960 Sierre, Switzerland; manfredo.atzori@hevs.ch (M.A.); henning.mueller@hevs.ch (H.M.); 3 Medical Faculty, University of Geneva, 1211 Geneva, Switzerland; 4 Institute of Intelligent Industrial Technologies and Systems for Advanced Manufacturing (STIIMA), National Research Council of Italy (CNR), 23900 Lecco, Italy

**Keywords:** muscle synergies, hand grasps, variability, intra-session, inter-session, virtual electrode repositioning

## Abstract

Background. Muscle synergy analysis is an approach to understand the neurophysiological mechanisms behind the hypothesized ability of the Central Nervous System (CNS) to reduce the dimensionality of muscle control. The muscle synergy approach is also used to evaluate motor recovery and the evolution of the patients’ motor performance both in single-session and longitudinal studies. Synergy-based assessments are subject to various sources of variability: natural trial-by-trial variability of performed movements, intrinsic characteristics of subjects that change over time (e.g., recovery, adaptation, exercise, etc.), as well as experimental factors such as different electrode positioning. These sources of variability need to be quantified in order to resolve challenges for the application of muscle synergies in clinical environments. The objective of this study is to analyze the stability and similarity of extracted muscle synergies under the effect of factors that may induce variability, including inter- and intra-session variability within subjects and inter-subject variability differentiation. The analysis was performed using the comprehensive, publicly available hand grasp NinaPro Database, featuring surface electromyography (EMG) measures from two EMG electrode bracelets. Methods. Intra-session, inter-session, and inter-subject synergy stability was analyzed using the following measures: variance accounted for (VAF) and number of synergies (NoS) as measures of reconstruction stability quality and cosine similarity for comparison of spatial composition of extracted synergies. Moreover, an approach based on virtual electrode repositioning was applied to shed light on the influence of electrode position on inter-session synergy similarity. Results. Inter-session synergy similarity was significantly lower with respect to intra-session similarity, both considering coefficient of variation of VAF (approximately 0.2–15% for inter vs. approximately 0.1% to 2.5% for intra, depending on NoS) and coefficient of variation of NoS (approximately 6.5–14.5% for inter vs. approximately 3–3.5% for intra, depending on VAF) as well as synergy similarity (approximately 74–77% for inter vs. approximately 88–94% for intra, depending on the selected VAF). Virtual electrode repositioning revealed that a slightly different electrode position can lower similarity of synergies from the same session and can increase similarity between sessions. Finally, the similarity of inter-subject synergies has no significant difference from the similarity of inter-session synergies (both on average approximately 84–90% depending on selected VAF). Conclusion. Synergy similarity was lower in inter-session conditions with respect to intra-session. This finding should be considered when interpreting results from multi-session assessments. Lastly, electrode positioning might play an important role in the lower similarity of synergies over different sessions.

## 1. Introduction

The way in which the central nervous system (CNS) controls and coordinates many muscles in voluntary movements is a longstanding question [1]. Muscle synergy (MS) analysis is an approach to understand the neurophysiological mechanisms behind the hypothesized ability of the CNS to reduce the dimensionality of muscle control [2], supported by experimental evidence [3]. The recorded electromyography (EMG) signals can be represented by linearly combining time-invariant muscle synergies, each activated by a distinct time-dependent coefficient waveform, achieved with matrix factorization algorithms [4]. Several other synergy models are available, such as space-by-time [5] or time-varying synergies [3]. Muscle synergies have been employed in various research fields: gait analysis [6,7], sports [8,9], robotics [10], as well as classification of patients with pathologies [11,12] and in neurorehabilitation [13,14,15].

Referring to neurorehabilitation, several efforts were made in previous studies to characterize the patients’ EMG patterns with muscle synergies. It was shown that lower motor function (reduced Fugl–Meyer assessment scores) might be coupled with a reduction in the number of available synergies [12,16]. Furthermore, there is evidence suggesting that composition of spatial synergies may change in poststroke patients compared to healthy people, especially in more impaired individuals [12,16,17], whereas time activations of spatial synergies are not significantly different from healthy individuals [7,18]. It is documented that the process of “tuning” or augmenting already existing abnormal synergies rather than the extinction of the abnormal synergies may underlie recovery [12]. In addition, there are studies suggesting that synergies may be clinically useful for diagnosis and treatment planning (e.g., poststroke [17,19]).

In this context, it was shown that several aspects related to the evaluation of motor recovery and the evolution of the patients’ motor performance may be quantified with muscle synergies, both in single-session and longitudinal studies. In fact, synergy-based assessments require extracting muscle synergies from data recorded in different experimental conditions and/or sessions and subsequently matching and comparing them for purposeful assessments. Thus, the results need to be interpreted considering the effect of intra-subject and inter-subject variability as well as intra-session and inter-session variability. Variability can be defined in this context as a set of similarity-based metrics to compare synergies across experimental conditions. Intra-session factors for variability include natural trial-by-trial variability of performed movements [5]. On the other hand, inter-session variability not only considers the natural variability normally found in performed movements but also may include other factors such as slightly different electrode positioning [20] and other intrinsic differences in subjects that can change in time (e.g., recovery, adaptation, exercise, etc.). Assessing whether these sources of variability may influence the similarity of the extracted synergies (i) within the same session, (ii) across sessions, and (iii) across subjects is crucial to provide reliable assessments. This knowledge can help in interpreting whether the inter-session variability found after clinical therapy falls within the natural, subjective variability or if it is due to other factors such as motor recovery (while other confounding factors, such as different electrode positioning, may affect the measure as well).

These challenges have only been partially addressed in literature. The effect of some of these sources of variability on extracted muscle synergies was already emphasized in several studies on healthy people, when assessing upper-limb intra- and inter-subject synergy composition similarity [5,21,22] or gait variability [23]. Several studies tried to quantify variability in various conditions, even in hardly comparable designs and topics. Different body segments and tasks were considered (including bench press in upper-limbs [20], level walking in lower-limbs [24], and a complex gymnastics motor task in upper and lower limbs [25]). Some studies analyzed differences between sessions (e.g., between two sessions [20]), some between subjects (6 healthy volunteers [24] and 9 gymnasts [25]), and some between different movement repetitions [24]. The analysis approaches also differed between studies. Taborri et al. [24] analyzed the number of synergies and the quality of the reconstructed data using intra-class correlation (ICC) and coefficient of variation (CoV) of both global variance accounted for (VAF) and VAF of each muscle. Kristiansen [20], Frere, and Hug [25] compared time activations from two sessions while spatial composition was kept constant and vice versa. These studies conclude that, in general, variability is found intra-session and intra-subject as well as inter-subject. However, much can be done to assess these sources of variability in more detail [5,20,22]. In the case of patient evaluations, it is expected that this variability is found as well and is even more prominent, since in general, neurological patient movements have a lower repeatability and larger heterogeneity at the kinematic and EMG level than those of healthy people [26,27].

Several aspects related to the variability found in muscle synergies can be analyzed in more detail to provide an assessment preparatory to rehabilitation applications. More specifically, this study aims at quantifying the sources of variability of muscle synergy composition that are related to inter-subject variability as well as intra-subject variability separated into intra-session (different movement repetitions) and inter-session (different acquisition sessions). We decided to assess these features first on a dataset of healthy participants under the assumption that, in each session, the motor capabilities of the subjects recruited were the same. The analysis was performed using as a testing scenario the comprehensive, publicly available hand grasp NinaPro Database (dataset number 6), featuring surface EMG measures from two EMG electrode bracelets, placed at the forearm level [28,29]. At the first level of our analyses, we aimed to determine whether there is a significant difference in the variability found at the intra-session and inter-session levels. These sources of variability can be analyzed under two perspectives: first, assessing variability in the metrics for signal reconstruction (such as reconstructed VAF [7,24,25,30] and number of extracted synergies [25,31]) and, second, assessing similarity of spatial composition of synergies [20,25]. For this aim, we adopted the spatial synergy model, which is a standard approach employed in the literature for assessing EMG patterns, able to capture variability across trials. Furthermore, we question how simulated, slight displacements in the positioning of the EMG electrodes might influence variability in spatial synergy similarity. This is especially interesting for multi-session assessments where electrodes have to be reapplied. The difference in electrode position might be due to the electrode fixation approach (e.g., standalone electrodes or “bracelets”) and even whether the same person was applying electrodes, complexity of muscles detection, etc. Simulating the repositioning of electrodes was achieved by virtually interpolating data from neighboring channels, as suggested in a previous work [32,33]. This approach allowed us to run several comparisons (described in the Methods section in more detail), including determining whether synergies from the same session but with different electrode positioning were significantly different. Second, we could test if a repositioning configuration of electrodes exists that can increase inter-session synergy similarity. The underlying assumption of this analysis was that, considering that the performed tasks were the same, one factor influencing the inter-session variability might be the difference in electrode positioning from session to session. Under these premises, slight rotation of the original data may affect the similarity of synergies and may reduce assessment error due to electrode placement. Finally, in order to assess generalization of synergies between people, inter-session synergy similarity was compared with the inter-subject similarities.

By analyzing robustness, stability and similarity of extracted muscle synergies under the effect of factors that may induce variability (including inter-session measurements, intra-session natural variability, and inter-subject differentiation), this work contributes to the knowledge available regarding the clinical usability of muscle synergies. The presented results are application specific, since electrode repositioning is simulated and constrained to a bracelet configuration of electrodes that was used for the NinaPro database. Nevertheless, through this methodology, we give a preliminary assessment of the possible influence of electrode position on the stability of muscle synergies in hand grasps, possibly estimating the effects of electrode movement and subject variability.

## 2. Materials and Methods

**Figure 1:**
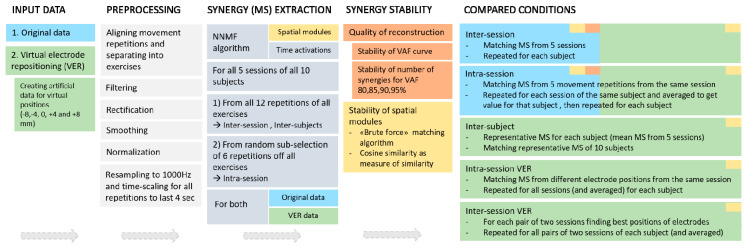
Overview of the steps of the data analysis that were performed. Two groups of input data were used: for the first part of the analysis, the original data from the NinaPro database (light blue background color) were used to quantify the difference in variability between intra-session and inter-session conditions; for the second part of the analysis, the artificially created data (green) were used to quantify effects of electrode repositioning. Preprocessing (white) was followed by synergy extraction (light gray) as described in detail in the text for both original and simulated datasets. Synergy stability was assessed using two approaches: variability in the metrics for reconstruction (variance accounted for (VAF) and number of synergies (NoS), red) and, second, assessing similarity of spatial composition of synergies (yellow).

A graphical overview of the data analysis performed in the study is shown in Figure 1. We considered two groups of input data: first, original data from the NinaPro database; second, artificially created data in order to test effects of Virtual Electrode Repositioning (VER). Steps for preprocessing of original EMG data as well as steps needed for synergy extraction are listed in Figure 1 and described in the Methods section. Synergy stability was assessed using two approaches: the variability in the metrics for reconstruction (using VAF and the number of extracted synergies) and, second, assessing similarity of spatial composition of synergies.

### 2.1. Data

**Figure 2:**
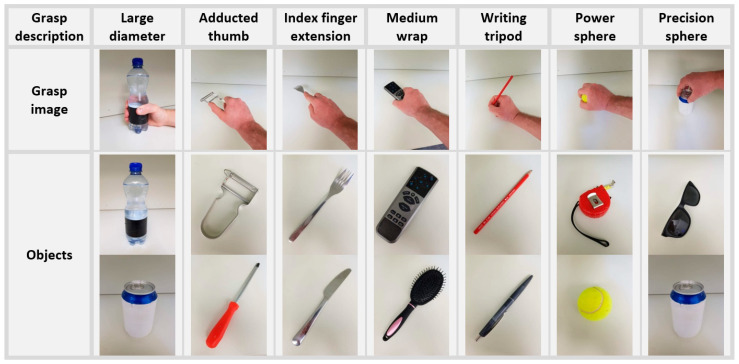
Seven daily-life grasps used in NinaPro DB6 (top row), each one employed for the grasping of two different objects (lower rows).

The NinaPro database [28] is available online with the aim to promote research on myoelectric hand prosthetics, motor control, and rehabilitation. It is the most extensive publicly available database in the field, containing multimodal data (surface EMG, CyberGlove data for kinematics of the hand, force measurement, eye-hand coordination data, etc.) from participants (healthy and amputees) performing over 40 different types of exercises [34]. The NinaPro database is divided into 10 datasets that differ in the movements performed, involved participants (number and type of subjects), and recording equipment. In this study, dataset 6 (Ninapro DB6) [29] was used. It contains data from 10 healthy individuals (3 females and 7 males, average age 27 ± 6) performing 7 grasp types shown in Figure 2, each repeated 12 times.

The set of hand grasps was chosen from the robotic and rehabilitation literature with the goal of covering several hand movements exploited in activities of daily living (ADL) [29,35,36]. The distinguishing feature of dataset 6 is that it contains data from participants who performed the same set of grasping movements for 5 consecutive days in 2 sessions per day. In order to avoid biases in the analyzed datasets such as adaptation related to daily repeated sessions or fatigue and to have equally time-spaced data, only the first session of each day was used in this study. Thus, the analyzed dataset considered 5 sessions for 10 subjects.

**Figure 3:**
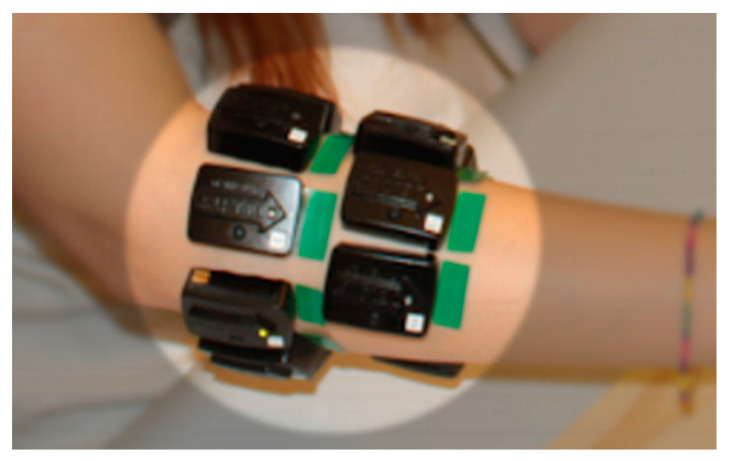
Positioning of EMG electrodes in the analyzed data (Ninapro DB6). Two arrays of electrodes were equally spaced on the forearm: the first array (in correspondence to the radio-humeral joint) included 8 electrodes, the second included 6.

**Figure 4:**
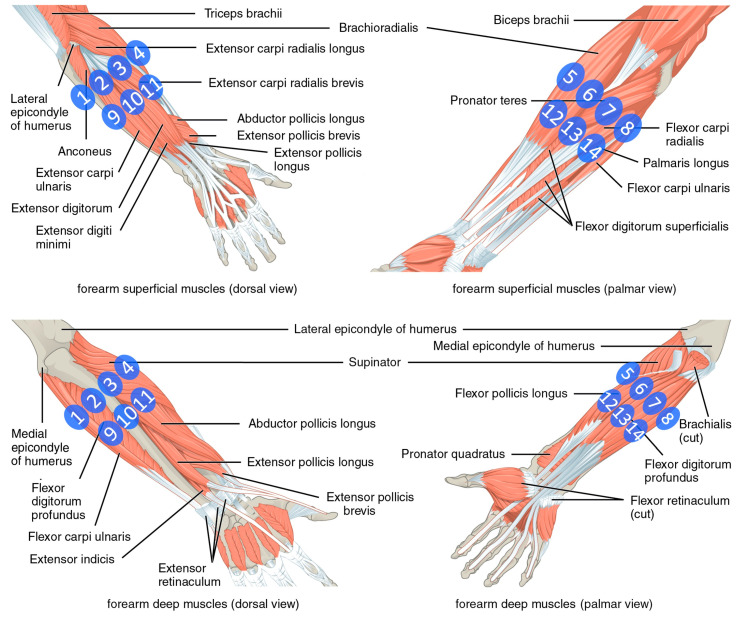
Positioning of the electrodes and underlying specific muscles. The image was adapted using file licensed under the Creative Commons Attribution 4.0 International license (Picture was adapted using https://upload.wikimedia.org/wikipedia/commons/7/73/1120_Muscles_that_Move_the_Forearm.jpg, from the Textbook OpenStax Anatomy and Physiology (source: https://cnx.org/contents/FPtK1zmh@8.25:fEI3C8Ot@10/Preface)).

Surface EMG was recorded with 14 Delsys Trigno sEMG wireless electrodes as shown in Figure 3. The electrodes were positioned around the forearm by the same experimenter using a dense sampling approach. Muscles underlying the electrodes are shown in Figure 4. Two rows of electrodes were fixed via bi-adhesive tape and covered with latex-free band to reduce their movement. The first row is composed of eight electrodes positioned sequentially and at equal distances starting from a landmark, the radio-humeral joint, identified by palpation as described in [28]. The second row is composed of six electrodes that are placed sequentially beneath the first row in correspondence to the empty spaces of the first row and avoiding positioning on the ulna. Dense sampling approaches are common in surface EMG literature as it was demonstrated that machine learning-based methods are insensitive to nominal electrode placement [37] and that they were also used in the past for studying muscular synergies [38]. Moreover, this setup based on uniform placement was already demonstrated to be effective [39], even on amputees [40], for sEMG signal classification and regression.

EMG data were sampled at 2000 Hz. The recorded signals correspond to the entire movements. The subjects were asked to sit in front of a table with the forearm leaning on it. The grasp to be executed was shown in two videos and then a set of audio indications explained the tasks to be performed (grasping the object, releasing the object, and returning to the rest position). A fixed image representing the grasp was shown on the screen of the laptop during the experiment. The number of repetitions was equally distributed among two different objects. Each repetition lasted for 4 s and was followed by 4 s of rest [29].

### 2.2. Data Analysis: EMG Processing and Synergy Extraction and Matching

#### 2.2.1. Preprocessing

The first step of data analysis was detecting the movement phases and aligning movement repetitions for each condition, grasp and subject considered. Moreover, data were preprocessed in several steps, with a preprocessing pipeline similar to the one employed in recent studies [6,41]. EMG data were band-pass filtered (20–400 Hz), rectified, and lastly smoothed with low-pass filtering with a 100-ms wide moving average window. The data were normalized using the maximal EMG value of each channel individually for each grasp type. Finally, data were resampled at 1000 Hz and rescaled in time so that all movement repetitions lasted exactly 4 s.

#### 2.2.2. Synergy Extraction

For each condition (e.g., session or sub-selection of movement repetitions) and subject, muscle synergies were extracted from concatenated EMG data of all exercises using nonnegative matrix factorization (NNMF) [42,43].

NNMF was repeated for a fixed number of extracted synergies ranging from 1 to the number of recorded EMG channels (14). Since NNMF is stochastic, analysis was repeated 50 times [24,44] with 100 iteration steps for each number of synergies. VAF was computed for each factorization order. The number of extracted synergies (NoS) was chosen as the lowest one explaining at least a predefined threshold level of VAF. Since different VAF thresholds were used in previous studies, we analyzed synergies that were extracted with 4 VAF thresholds equal to 80, 85, 90, and 95%.

#### 2.2.3. Synergy Matching

In order to compare synergies from different conditions (e.g., sessions, people, etc.), we set up a brute force algorithm enabling to produce meaningful synergy matching after NNMF [45]. The algorithm assessed all possible combinations of groups of synergies from different conditions. For each group, synergy similarity was calculated and synergies were sorted by highest similarity. Cosine similarity was used as a measure of synergy similarity [45,46,47], where 1 represents synergies with the same spatial composition and 0 represents orthogonal synergies. As a measure of similarity for each group, we used the average of all pairwise similarities of all pairs of two synergies from the group. When comparing synergies from n sessions, each group consisted of a maximum of n synergies or fewer if the number of synergies extracted from different sessions was not identical (due to different VAF curves). After similarity scores were calculated for all possible combinations of groups, the algorithm chose the group with highest similarity and removed all combinations (groups) that contained already used synergies. Then, it chose the next best combination and continued until there were no more synergies left to pair. In the end, the algorithm provided the average similarity of final clusters of synergies (matching score). The average cosine similarity of all possible combinations of synergy groups was also measured (random score) to be sure that matching was indeed performing functional sorting.

### 2.3. Data Analysis: Considered Cases

#### 2.3.1. Intra-Session and Inter-Session

**Figure 5:**
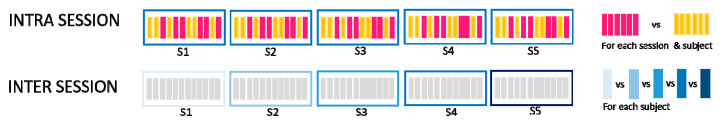
Two data groups were identified to perform analysis of muscle synergy stability. The intra-session dataset grouped synergies were extracted from 5 subsets of 6 repetitions of movements that was repeated for each session and subject. Here, for simplification, only 2 subsets are shown. The inter-session dataset grouped all 12 movement repetitions within each session to extract synergies representing a session. Five synergy sets (of 5 sessions) for each subject were compared.

We classified several aspects of synergy stability and robustness, which we achieved by decomposing the NinaPro dataset as illustrated in Figure 5, where data structures are illustrated, including the following groups for analysis.

Intra-session analysis focused on a comparison of synergies that were extracted from movement repetitions within the same session. Synergies were extracted from 6 randomly selected repetitions of movements out of the 12 available that were recorded in each session (see Figure 5). Extraction of synergies, preceded by random selection of 6 repetitions, was repeated 5 times for each session and subject. Comparison between 5 extracted synergy sets from the same session followed. This was repeated for all sessions and subjects.

Inter-session analysis focused on comparing metrics related to extracted synergies from different sessions. To achieve this result, all 12 repetitions of movements from the same session were concatenated and used to extract synergies of that session. The synergy sets of 5 sessions of each subject were compared (see Figure 5).

#### 2.3.2. Comparison of Synergies from Virtual Electrode Repositioning

The aim of this analysis was to assess the role of electrode positioning as a source of difference between inter-session and intra-session similarity of synergies. Placing electrodes in a slightly nonrepeatable way between sessions can happen in real scenarios and has been mentioned already as a possible reason for lower inter-session similarity and stability of synergies with respect to intra-session assessments [20].

The NinaPro dataset 6 (Ninapro DB6) is a valuable resource to study the effect of electrode positioning on muscular hand synergies, and to our knowledge, no other publicly available datasets include the repetition of the same electromyography acquisition protocol in different sessions. The NinaPro DB6 contains recordings performed during 10 acquisition sessions with the EMG electrodes positioned in a bracelet configuration. The electrode configuration allows to simulate slight electrode displacements by artificially calculating values of EMG data from the recorded channels. The electrodes in each bracelet were equally spaced, and the bracelets were positioned according to anatomical landmarks. A first approximation to simulate the misplacement of the electrodes is to assume that each electrode in the same bracelet was rotated medially or laterally by the same distance. Thus, expected differences in rotation could be due to the possibility that the electrodes were misplaced by a few millimeters to the medial or lateral side according to the anatomical landmark. Again, a preliminary approximation to simulate the misplacement of the electrodes could be to assume that the misplacement can be approximated by linear interpolation of the EMG activity from adjacent electrodes. Similarly, in fact, each Delsys Trigno electrode detects sEMG at the skin surface by 4 silver bar contacts that are 5 mm large and positioned a few millimeters from each other. Obviously, this approach is a strong simplification of the electrode misplacement problem. The real change in the recorded muscle activity is likely nonlinear due to muscle physiology, as well each electrode is likely moved singularly further or closer to the anatomical landmark. Nevertheless, the simplification is justified by the limits of the acquisition setup itself (the size of the electrodes and the silver bars being comparable in order of magnitude to the virtual displacement), and it was sufficient for the aim of this analysis (i.e., to experimentally evaluate how much slightly different positions of electrodes can influence the similarity among synergies).

**Figure 6:**
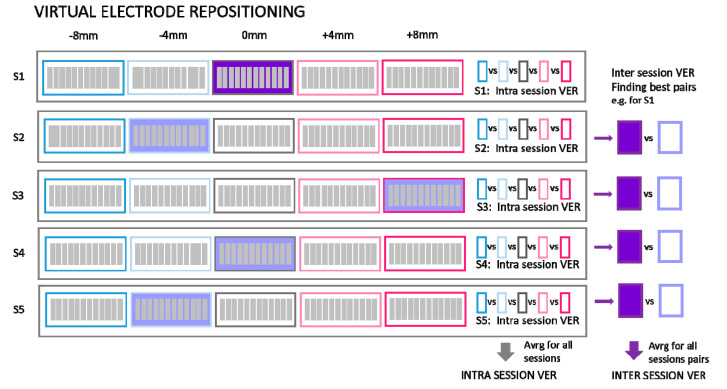
Summary of the composition of the datasets to analyze the possible influence of electrode repositioning: For each session (S1–S5) of each subject, data for 4 simulated displacements (−8 mm, −4 mm, 4 mm, and 8 mm) were calculated interpolating data from neighboring electrodes. This new artificial data was used (1) to calculate similarity of extracted synergies between steps from the same session (intra-session VER) and (2) to calculate similarity of extracted synergies between steps from different sessions (inter-session VER). This was achieved by calculating and comparing similarities of synergies between all combinations of steps between two sessions to find the optimal step for each session related to the reference session (in the figure, we assumed S1 as the reference). This optimal step can represent a reconfiguration of the positioning corresponding to the most similar positioning with respect to S1. The same procedure was repeated considering each session as reference and calculating the average of all max similarities (inter-session VER).

Five simulated step sizes were tested (Figure 6) according to the following rationale. The database 6 includes information about the forearm circumference of each participant, so we can estimate average space between two consecutive electrodes. The distance between the centers of two electrodes was from 27.5 mm to 38.75 mm depending on the subject. Since the width of Delsys Trigno electrodes is 27 mm, the inter-electrode distance was between 0.5 mm and 11.75 mm. Consequently, we chose to simulate displacement as a tradeoff between computational complexity and realistic electrode displacement: 8 mm and 4 mm displacements both to the medial and lateral directions as well as center position were thus considered in the following analysis.

During the recordings of Ninapro DB6, the equipment consisted of two electrode bracelets; both of them could, in theory, be moved for different step sizes or even in different directions. It follows that the number of possible movement combinations exponentially increases. Thus, at the present stage, we decided to limit our analyses to the more proximal bracelet including only the first 8 electrodes (closer to the radio-humeral joint) and assume that all 8 electrodes were moved by an equal step size in the same direction.

**Figure 7:**
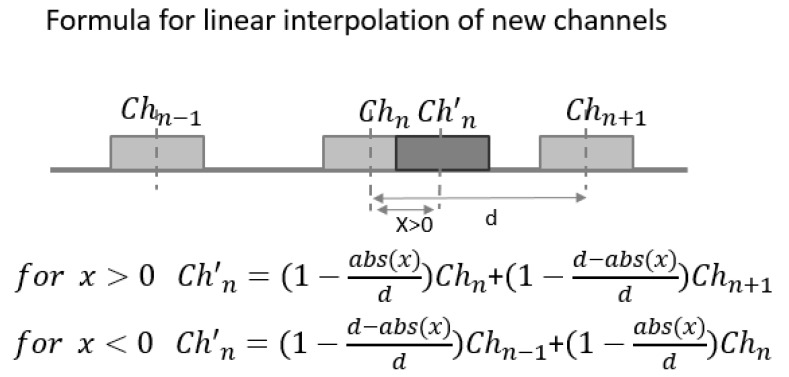
Linear interpolation for simulating new EMG channels: Depending on the direction of the electrode displacement, relevant neighboring channels were used. New artificial EMG data were estimated by linearly summing up data of neighboring channels multiplied with factors that are reverse proportional to distances, as proposed in recent studies (see text).

New artificial EMG data were created by linearly interpolating EMG recordings from two neighboring electrodes using the formula and schematics shown in Figure 7. This approach was inspired by the work of Atzori et al. [32]. We observe that the artificially calculated EMG data and virtual movement of electrodes do not include any physiological information. We are aware that this is a simplified model approach and we do not claim that these exact data would have been recorded if the electrodes were really in these positions. Similar approaches were used to compensate pattern recognition failures due to electrode shift in literature [33]. Despite these limitations, our simulated scenario can add valuable knowledge about how relevant the positions of the electrodes are for the stability of muscle synergies. All datasets achieved with simulated data were labelled with “VER” suffix (Virtual Electrode Repositioning).

The analysis was performed as follows: (1) From the original data, new artificial data were created by interpolating new channels for steps of −8 mm, −4 mm, 0 mm, 4 mm, and 8 mm. This simulation was performed for each participant and each session. (2) For each electrode displacement step, synergies were extracted with NNMF from all steps of all sessions of each participant. (3) Matching was performed between extracted synergies. Depending on the purpose of the analyses, synergies were matched according to specific criteria (either same session but different steps, IntraSessVER, or different sessions with different steps, InterSessVER) as shown in Figure 6. Combinations of pairs of synergies from different sessions and steps were matched optimally using the brute force matching as described above.

### 2.4. Data Analysis: Outcome Measures

#### 2.4.1. VAF and Number of Extracted Synergies

First, we computed variance accounted for (VAF) curves depicting how VAF changes, when an increasing number of synergies was extracted with the NNMF algorithm for different sessions (inter-session) and different sub-selections of movements for the same sessions (intra-session). VAF values were used as a measure of the quality of reconstruction of the data. Furthermore, we analyzed the number of synergies (NoS), which was also employed as a measure of synergy stability [24]. Since in the literature, there is no consensus on the threshold for the VAF and which VAF threshold is high enough [48], in this study, we present results related to four VAF thresholds that were adopted in the literature (80, 85, 90, and 95%).

In order to quantify variability of VAF and NoS among sub-selections of movements or between sessions, the coefficient of variation (CoV) was used. It is a measure of the dispersion of data points around the mean. Subsequently, CoV was calculated for inter-session and intra-session VAF curves and NoS values. The inter-session dataset was created by computing the VAF (and NoS) values for each of the 5 sessions of each subject, whereas intra-session dataset was achieved by computing the VAF (and NoS) values for 5 movement sub-selections (containing 6 movement repetitions) inside the same session (and repeated for all sessions and subjects). To get representative intra-session CoV for each subject, 5 CoV from 5 sessions were averaged. In this way, our dataset was composed of 10 CoV for VAF values (one per subject) for each number of extracted synergies (1 to 14) and for each experimental condition (intra and inter) as well as 10 CoV for NoS values (one per subject) for each VAF value and for each experimental condition (intra and inter). In order to investigate the difference between the amount of variability intra-session compared to inter-session condition, the difference in the CoV values was assessed using two-way repeated measures ANOVA using condition type (inter and intra) and VAF (or NoS) as factors.

#### 2.4.2. Similarity of Synergy Spatial Composition

Synergies in intra- and inter-session conditions were compared also according on their spatial composition.

The inter-session synergy similarity assessed how similar is the spatial composition of synergies between different sessions in which an individual performed the same sets of movements. EMG data were extracted and filtered from each session individually, followed by the matching of synergies from 5 sessions. The most similar synergies between days, found with the “brute force” matching algorithm (described in Section 2.2.3), were grouped under the assumption that they represent the same synergy and how it changed over days. For each group of matched synergies, the average pairwise cosine similarity was reported. To get representative inter-session similarity values, the average similarity of all matched groups was calculated for each subject. In this way, our inter-session dataset for spatial synergy similarity was composed of 10 average matching values (one per subject) for each VAF value.

The intra-session synergy similarity assessed how similar the spatial composition of synergies is when using data recorded during the same session but in different movement repetitions. Similar to inter-session, brute force matching of 5 muscle synergy sets (from each sub-selection of movement repetitions) was performed. Analysis was repeated for each session and each participant. Intra-session similarity of each subject was determined as the average of intra-session similarities of all 5 sessions for this subject. In this way, our intra-session dataset for spatial synergy similarity was composed of 10 average matching values (one per subject representing average of 5 sessions) for each VAF value. Since the aim of these analyses was to investigate the relationship between the amount of variability due to intra-session variability and inter-session variability, we tested our dataset to determine the difference in the average spatial synergy matching values between the two conditions (inter and intra) using condition and VAF as factors.

#### 2.4.3. Virtual Electrode Repositioning

**Figure 8:**
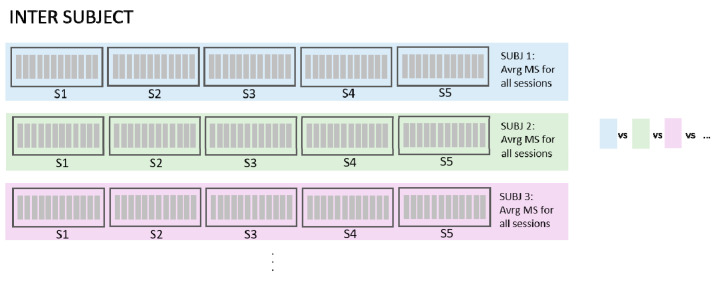
Data structure and analysis steps used for inter-subject analysis: Synergies of all 5 session of each subject were extracted, optimally matched, and averaged to achieve representative synergies for each subject. Representative synergies were then compared (matched) between subjects. Inter-subject similarities were later compared with inter-session similarities in order to test generalization of synergies between subjects in Comp#5.

In order to assess the effect of slight electrode repositioning on variability in synergy similarity and since the exact position of EMG arrays on the person’s skin was not available, we created a set of simulated conditions (virtual electrode repositioning). The following datasets were considered:

1. Intra-session virtual electrode repositioning (IntraSessVER)
  The first step for our analysis was to investigate if a different synergy similarity is detected when slightly repositioning electrodes within the same session. This finding can confirm that electrode repositioning might influence synergy similarity and serves as motivation for the following steps of the analyses.
  To achieve this, we created a simulated dataset to compare muscle synergy similarity from different virtual electrode positions for the same session. We compared average similarities for all possible repositioning distances (4 mm, 8 mm, 12 mm, and 16 mm). This was repeated for all VAF values. Maximal similarity of synergies (MAX) between any two steps of rotations for each session (and subjects), average similarities (AVRG) between all step pairs, and minimal similarities (MIN) were calculated. MAX values represented, for each subject, the average (computed across 5 sessions) of maximum similarities detected between different electrode positions within each session. To achieve AVRG value similarity, values of all step pairs in each of the sessions were averaged (for each subject). Finally, MIN values represented the average (computed across 5 sessions) of minimum similarities for each subject detected for any combination of electrode positions within same session. In this way, our IntraSessVER dataset was composed of 10 AVRG, 10 MIN, and 10 MAX matching values (one for each subject representing an average of 5 sessions) for each VAF value.
2. Inter-session virtual electrode repositioning (InterSessVER)
  Here, we aimed to assess if repositioning electrodes virtually between sessions can lead to higher similarity between sessions. If this condition was met, a part of the variability in the muscle synergy similarity that existed between sessions can be due to slightly different positions of electrodes. For each possible combination of two sessions, we created a simulated dataset computing the maximum muscle synergy similarity by calculating all combinations of steps for those two sessions. Maximum, minimum, and average similarities between different step pairs (25 combinations) and session pairs (10 combinations) were calculated for all the subjects. MAX values represented the highest similarity that each subject achieved between any two sessions having the option to also shift electrodes between sessions. MIN values reported the minimum value that each subject had between any two sessions. AVRG values were the average of MAX values found for each session pair, calculated for each subject. In this way, our InterSessVER dataset was composed of 10 ARVG, 10 MIN, and 10 MAX matching values (one for each subject) for each VAF value.
3. Intra-session without electrode repositioning (IntraSessORIG)
  The intra-session dataset was created by computing the similarity of the synergies extracted from a different selection of movement repetitions but from the same session (no electrode repositioning). This was the same analysis as already described in Section 2.4.2, but it was repeated using only 8 channels instead of 14 in order to be able to compare the results with that of intra-session using virtual electrode repositioning. Minimum, maximum, and average values of 5 sub-selections of movements from each session were calculated. Then, the average of minimum, maximum, and average values over 5 sessions for each subject were compared with values of the other conditions. MAX values represented for each subject the average (computed across 5 sessions) of maximum similarities detected between movement sub-selections, AVRG values represented the average of average similarities between sub-selections for all sessions, and the MIN average represented minimum similarities of its sub-selections. In this way, our IntraSessORIG dataset was composed of 10 AVRG, 10 MIN, and 10 MAX matching values (one for each subject representing average of 5 sessions) for each VAF value.
4. Inter-session without electrode repositioning (InterSessORIG)
  The intersession dataset was created by computing the similarity of synergies between sessions. The dataset was analogous to the one described in Section 2.4.2 but for only 8 channels instead of 14 and with the original positions of electrodes (no electrode repositioning). MAX values represent the maximum similarity that each subject achieved between any two sessions (with original positions of electrodes), while MIN values report the minimum value that each subject had between any two sessions. AVRG values are the average of all possible session pairs (10 combinations) for each subject. In this way, our InterSessORIG dataset is composed of 10 AVRG, 10 MIN, and 10 MAX matching values (one for each subject) for each VAF value.
5. Inter-subject (InterSUBJ)
  Finally, we considered a fifth condition that we labelled inter-subject (portrayed graphically in Figure 8). The assessment of similarity of spatial synergies between people can enhance the understanding of the generalization of the synergies. The average synergies computed across 5 sessions for each person were calculated (using groups from inter-session matching described in Section 2.4.2) and averaged in the view that they represent individual synergies better than if we chose synergies from one session randomly. These average synergies, representing individual synergies of 10 participants, were then compared in a pairwise manner (45 combinations). MAX values represent the maximum similarity of synergies that each subject had with one of the other subjects (9 combinations), AVRG values are average similarities that each subject had with all other subjects, and MIN values represent the minimum value of similarities each subject had with any of all other subjects. In this way, our InterSUBJ dataset is composed of 10 AVRG, 10 MIN, and 10 MAX matching values (one for each subject representing the average of comparison with 9 other people) for each VAF value.

Given these 5 sub-datasets, the following comparisons were analyzed (graphically portrayed in Figure 16, as an example for VAF = 80%):

Comp#1: to test whether significant differences existed between similarities of synergies extracted from the same sessions (IntraSessORIG) and synergies from different sessions (InterSessORIG) both without virtually repositioning of electrodes. This was analogous to the comparison in Section 2.4.2 but with 8 electrodes instead of 14.
Comp#2: to test whether a significant difference exists in average values of similarities between intra-session (IntraSessORIG) and intra-session with different steps in virtual electrode repositioning (IntraSessVER). This allowed to show whether higher, lower, or comparable intra-session synergy similarity was achievable when electrodes are virtually repositioned.
Comp#3: to test whether a significant difference exists between the average value of similarities of sessions with original positions (InterSessORIG) and sessions with the best electrode positioning combination (InterSessVER). This allows us to test whether on average it is possible to get higher inter-session similarity when allowing electrode displacement between sessions. In other words, is it possible that slight electrode repositioning between sessions is one of the factors influencing lower synergy similarity?
Comp#4: to test whether a significant difference exists between maximal similarities that can be achieved for inter (InterSessVER) and intra-session (IntraSessVER) conditions while allowing electrode repositioning. This allows to test if, by moving electrodes between sessions, for any combination of sessions, it is possible to get similarity as high as with intra-session data.
Comp#5: to test whether a significant difference exists between synergies of the same subject extracted in different sessions (InterSessORIG) when compared to synergies of other subjects (InterSUBJ). This investigation tests how well muscle synergies generalize between individuals with respect to the average inter-session similarity. The analysis was done in order to assess whether lower inter-session (with respect to intra-session) similarities impose serious concern for multi-session or longitudinal assessments. If inter-subject similarities were lower than inter-session ones, synergies contained subject specific information, but generalization of synergies between subjects may not be very high. On the other hand, if inter-subject similarities were on the same level (or higher) than inter-session, then the synergies generalize well between subjects but it is not possible to distinguish whether synergies are from the same subject but recorded during different sessions or from a completely different subject. This can be a serious point to consider for multi-session assessments.

For all the proposed objectives, the dataset was composed of 10 values for MAX, AVRG, and MIN value (one for each subject) for each VAF and condition. All the comparisons were repeated for 4 VAF levels: 80, 85, 90, and 95%.

### 2.5. Statistical Analysis

Two-way repeated ANOVA measures were applied in the analyses. The used factors are the condition of the analysis (e.g., inter-session vs. intra-session) and VAF or NoS that were used as a criterion for extraction of muscle synergies. Mauchly’s test for sphericity was conducted, and *p*-values were corrected if sphericity was not satisfied. All tests were performed in the statistical software R [49] (version 3.6.1).

## 3. Results

### 3.1. Intra-Session and Inter-Session Variability

#### 3.1.1. VAF and Number of Extracted Synergies

**Figure 9:**
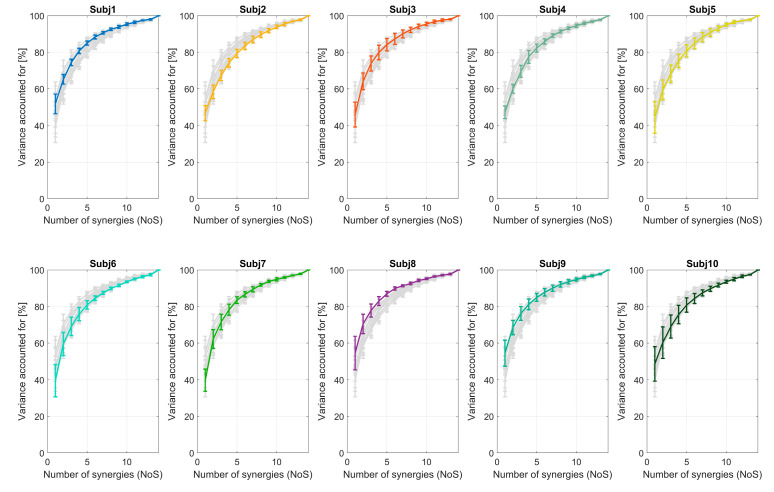
For each of the ten subjects, the amount of reconstructed VAF is portrayed against the number of extracted synergies. In each subplot, we portray one subject (colored line) compared to all the other 9 subjects (gray lines). Mean and standard deviation are shown representing variability between the VAF values of 5 sessions of the same subject.

**Figure 10:**
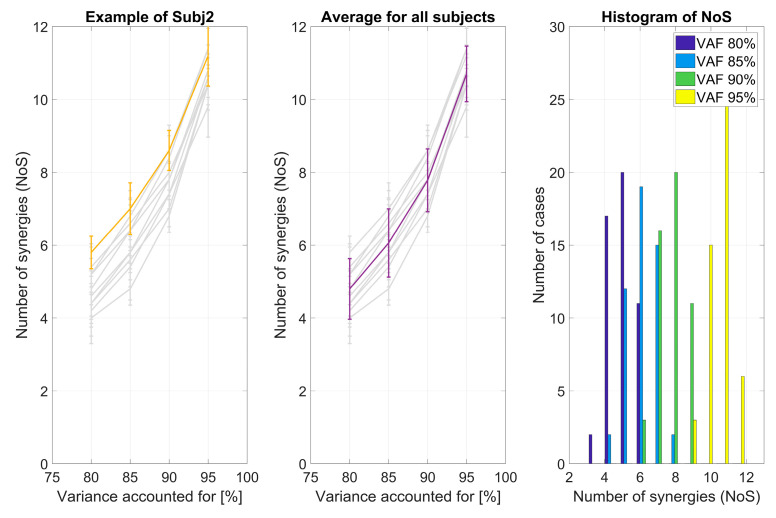
For each subject, the NoS is portrayed for each of the four selected VAF thresholds and how NoS varies between five considered sessions. The example of the mean and standard deviation of the NoS for one typical subject (Subj 2) in comparison with all the others is shown (**a**) as well as total mean and STD for all subjects and sessions (**b**). For each of the four VAF levels, a histogram portrays the number of cases in which a specific number of synergies was extracted. The higher the threshold, the higher the average number of extracted synergies (**c**).

The VAF curve varied between sessions for each subject. This is portrayed in Figure 9, where each subplot shows the VAF curve for each of the 10 subjects (colored line) with respect to the others (light gray lines) and error bars represent the standard deviation among sessions for each subject. The number of synergies (as it changes through sessions using four different VAF thresholds) was analyzed and is shown in Figure 10. In Figure 10, we show (a) the results obtained for one representative subject; (b) the average for all the subjects; and (c) a histogram of the chosen number of synergies for different VAF thresholds and all sessions and subjects. The number of synergies ranged, for VAF 80%, from 3 to 6 (most commonly 5); for VAF 85%, from 4 to 8 (most commonly 6); for VAF 90%, from 6 to 9 (most commonly 8); and, for VAF 95%, from 9 to 12 (most commonly 11). The differences in the number of synergies are not neglectable, and they seem to overlap between subjects (Figure 10b).

The same analysis based on repeatability of the VAF curve and representative NoS was repeated for the intra-session approach for the four different VAF thresholds. The VAF curve and the NoS were compared between sub-selections of movement repetitions from the same sessions.

**Figure 11:**
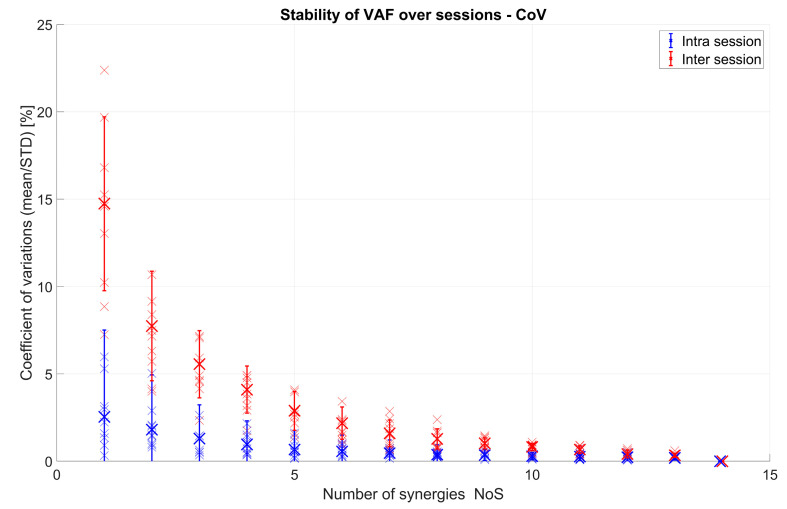
Intra-session (blue) and inter-session (red) coefficient of variation (CoV) of VAF values for 10 subjects: Inter-session was achieved by comparing VAF values between 5 sessions of each person, whereas intra-session was achieved by comparing VAF values for 5 movement sub-selections inside the same session (and repeated and averaged for all sessions). Each small “x” represents one person, and the bigger “X” represents the mean of all 10 subjects.

VAF and NoS vary between sessions and even for different movement repetition selections (not shown). In order to assess significance of this variation and to quantify the difference between inter- and intra-session variation, the coefficient of variation (CoV) was used. In Figure 11, the CoVs of VAF values for both inter-session and intra-session analysis are shown. It can be seen that VAF values varied on average within less than 3% for intra-session analysis and up to 15% for inter-session analysis. The difference between intra-session and inter-session comparison was statistically significant (F(1,9) = 68.33, *p* < 0.001). The number of synergies also influenced stability (CoV) of VAF values (F(13,117) = 83.14, *p* < 0.001). The interaction between the type of comparison (inter vs. intra) and the number of synergies was also significant (F(13,117) = 48.28, *p* < 0.001).

**Figure 12:**
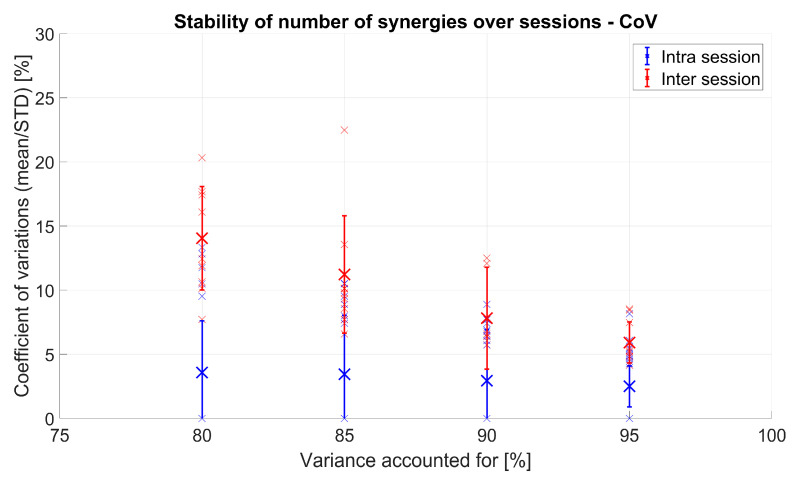
Intra-session (blue) and inter-session (red) CoV of NoS values for 10 subjects: Inter-session was achieved by comparing NoS values between 5 sessions of each person, whereas intra-session was achieved by comparing NoS values for 5 different movement sub-selections inside the same session (and repeated and averaged for all sessions). Each small “x” represents one person, and the bigger “X” represents the mean of all 10 subjects.

Figure 12 shows the CoV values of NoS that are needed to represent 80, 85, 90, and 95% of the variance of the original EMG data. NoS values for intra-session comparison varied on average by less than 4%, while inter-sessions had higher values of up to almost 15% on average for all subjects. A significant difference between intra- and inter-session was found (F(1,9) = 45.99, *p* < 0.001). Similarly, VAF (F(3,27) = 13.71, *p* < 0.001) and interaction (F(3,27) = 10.8, *p* < 0.001) effects were also significant.

Both from Figure 11 and Figure 12, it is visible that the CoV difference is more emphasized when the VAF and the NoS are lower.

#### 3.1.2. Spatial Muscle Synergy Similarity

**Figure 13:**
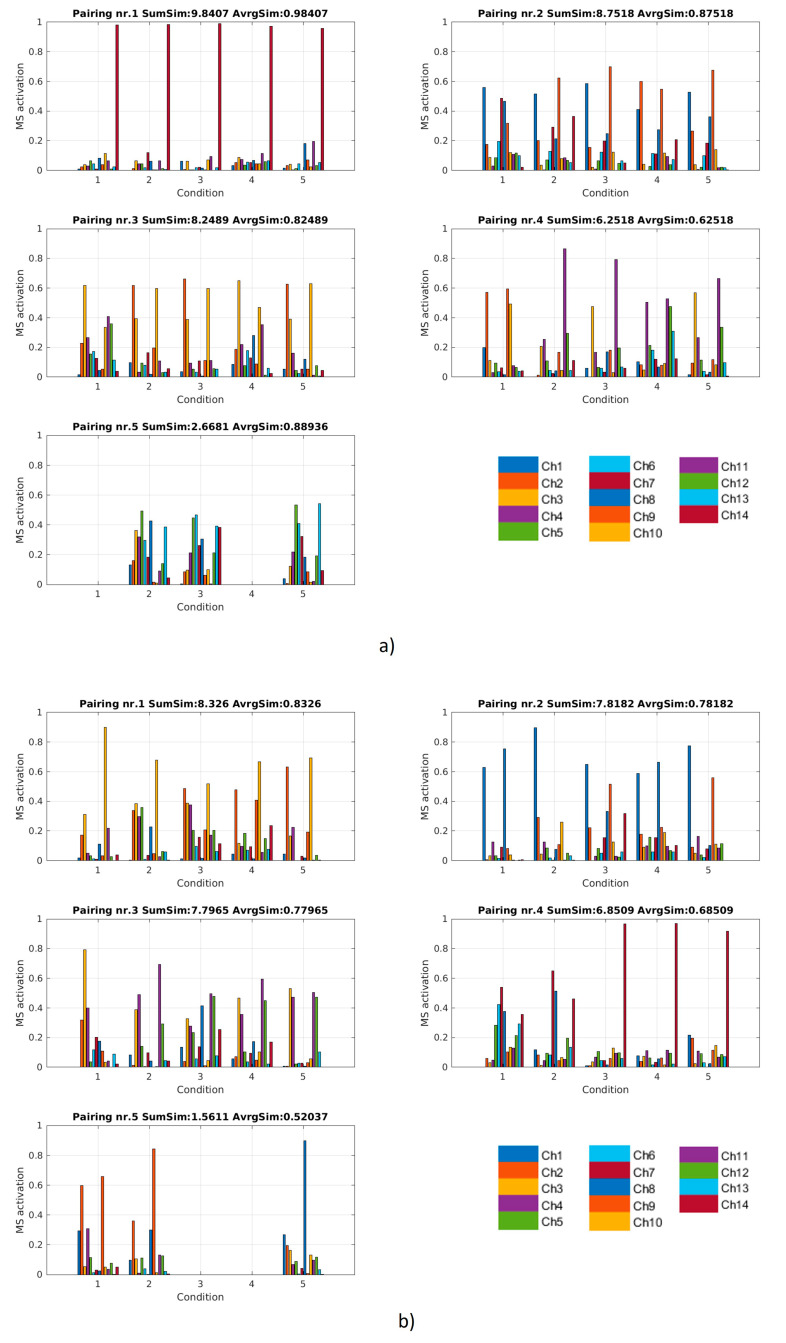
A typical person with a high inter-session matching score (**a**) and lower inter-session matching score (**b**): In both cases, a VAF of 85% was considered. The X axis represents five sessions from which synergies were extracted. On the top-left graph, five synergies (one for each session) are shown and they represent a group of the five synergies with the highest similarity, chosen from the pool of synergies extracted for all five sessions. The groups of synergies in each subplot show the result of optimal matching of synergies. In the titles, the average similarity and the sum of all pairwise similarities in a given matching group (cluster) is reported. Clusters were sorted based on the sum of similarities (SumSim).

In Figure 13, the matching of synergies from five sessions is shown for two subjects. The figures present results for a participant with high average similarity and one with low similarity of synergies among sessions in order to qualitatively show what similarity values between approximately 0.5 to approximately 0.9 look like, when visually comparing synergies. The results of the optimal matching strategy as described in Section 2.2.3 were groups (clusters) of up to five synergies (each shown in a different subplot). Clusters were sorted based on average similarity of synergies within it (reported in the title of each subplot). The value of inter-session similarity for each subject was calculated as average of similarities of each cluster for this subject.

**Figure 14:**
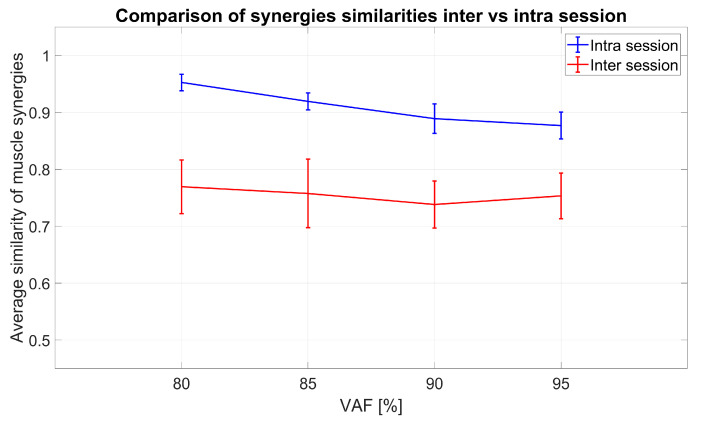
Inter-session (red graphs) and intra-session (blue graphs) similarity values for several VAF thresholds, summarized for all 10 subjects: Inter-session represents similarities between sessions of the same subject, while intra-session similarity between synergies is achieved comparing several sub-selections of movements (from the same session) across 5 sessions for each subject.

In Figure 14, the results of matching of synergies between several sessions for all 10 subjects and four VAF criteria are shown (red). Average similarities between sessions after optimal matching with the described “brute force” algorithm ranged from 0.7 to 0.85 depending on the subject and VAF. Analogous to analysis of inter-session synergy, synergies from several selections of movement repetitions were computed and averaged to determine intra-session similarity. In Figure 14, the scores for optimal matching of intra-session are shown in blue. Intra-session values are significantly higher than inter-session values for all VAF (F(1,9) = 276.73, *p* < 0.001). The VAF also has significant influence (F(3,27) = 7.34, *p* < 0.001) while interaction after sphericity correction is not significant (F(3,27) = 2.49, *p* = 0.12).

### 3.2. Virtual Electrode Repositioning

**Figure 15:**
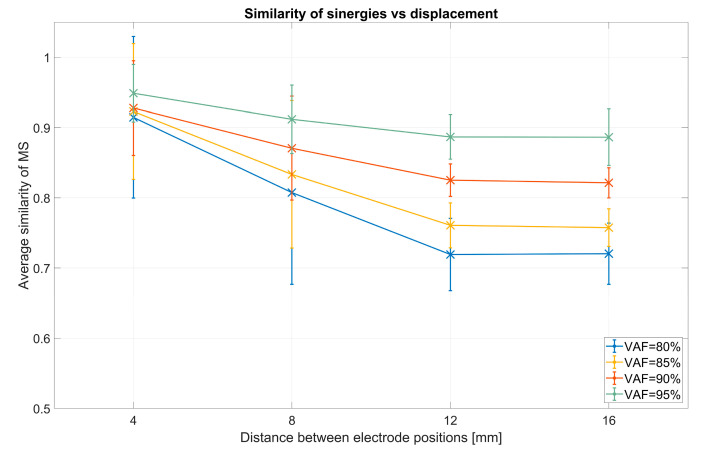
Intra-session muscle synergy (MS) similarity plotted against distance between electrodes: The graph clarifies the decreasing trend in synergy similarity when simulating increasing distance between electrodes.

In order to investigate why the inter-session similarity is significantly lower than intra-session similarity, a possible influence of electrode positioning was tested by simulating data from slightly different positions of the electrodes. Results are shown in Figure 15. Similarity drops significantly (F(3,27) = 669.33, *p* < 0.001) after sphericity correction, as the distance is increased and stabilizes with bigger distances. There is also significant influence of VAF (F(3,27) = 37.74, *p* < 0.001) after sphericity correction and an interaction between VAF and distance (F(3,27) = 37.82, *p* < 0.001) after sphericity correction.

**Figure 16:**
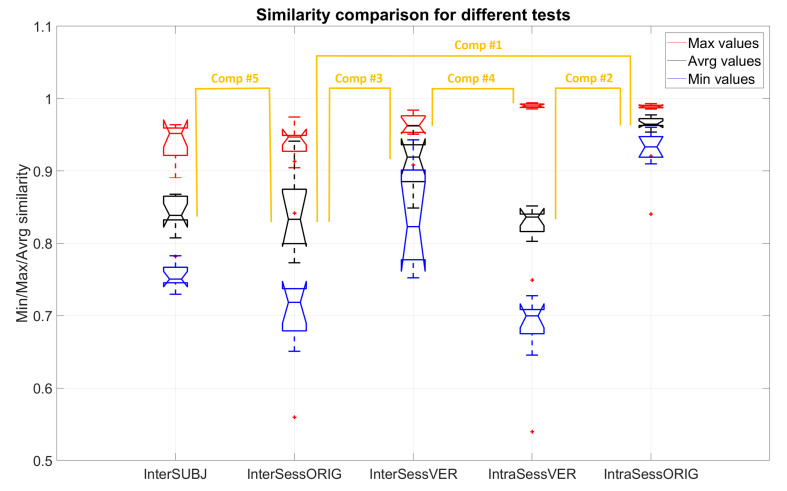
Comparison of muscle synergy similarity with different analysis approaches: inter-session (InterSessORIG), intra-session with sub-selection (IntraSessORIG), intra- and inter-session with virtual electrode repositioning (IntraSessVER and InterSessVER), and inter-subject (InterSUBJ) for all 10 participants and VAF threshold = 80%. The X axis represents different tests as described above, and Y axis represents MIN (blue), MAX (red), or AVRG (black) values of similarities. The details of each test are reported in the text.

**Figure 17:**
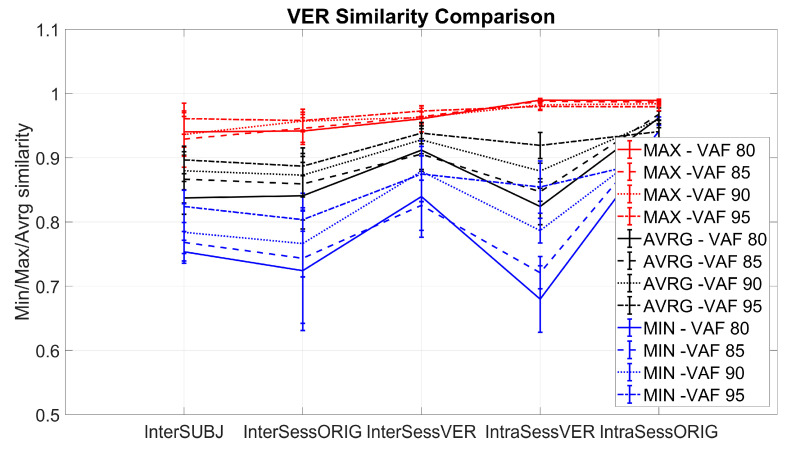
Comparison of muscle synergy similarity in the five considered conditions: inter-session (InterSessORIG), intra-session with sub-selection (IntraSessORIG), intra- and inter-session with virtual electrode repositioning (IntraSessVER and InterSessVER), and inter-subject (InterSUBJ) for all 10 participants and several VAF thresholds. The X axis represents several tests as described above, and the Y axis represents MIN (blue), MAX (red), or AVRG (black) values of similarities.

Figure 16 portrays the results of 5 comparative analyses using the approach of virtual electrode repositioning, shown for VAF threshold = 80%. Results for all the VAF thresholds for all the mentioned analyses are shown with a synthetic layout in Figure 17. In both figures, the X axis lists the analysis types that were performed and the Y axis shows MIN (blue), MAX (red), or AVRG (black) values of muscle synergy similarity for each of the conditions. MIN, MAX, and AVRG values were defined for each dataset as explained in Section 2.4.3.

The comparison of the similarity between different conditions and how they relate to each other is given as an example for a VAF value of 80%. For inter-session (InterSessORIG), average values (black) of synergy similarity between pairs of sessions were 0.841 ± 0.052, maximal values (red) of similarity between two sessions for different subjects were 0.942 ± 0.021, while minimal values (blue) were 0.722 ± 0.093. For intra-session (IntraSessORIG), these values were much higher, all above 0.9 (maximal 0.989 ± 0.002, average 0.962 ± 0.015, and minimal 0.927 ± 0.032). Average similarities of synergies between two people (InterSUBJ) were 0.837 ± 0.026. The highest similarity between two subjects was 0.940 ± 0.024. The lowest similarity between two subjects was 0.754 ± 0.018.

When assessing changes in similarity, when electrodes were virtually moved within the same session (IntraSessVER), the highest similarity (for the given 5 steps) was 0.989 ± 0.003, while average values for different combinations of the steps were 0.824 ± 0.028 and minimal ones were 0.680 ± 0.052. When assessing whether similarity can be increased between sessions by comparing synergies from various positions (InterSessVER), the highest values obtained were 0.961 ± 0.020, average values were 0.912 ± 0.033, and minimal ones were 0.839 ± 0.063.

Comparing inter-session (InterSessORIG) and intra-session (IntraSessORIG) similarities without option to reposition electrodes (reported in Figure 16, Comp#1), revealed similar results as can be seen in Figure 14. A significant difference between inter-session and intra-session conditions was detected (F(1,9) = 83.05, *p* < 0.001) but without influence of VAF (F(3,27) = 0.94, *p* = 0.4 after sphericity correction) and significant interaction (F(3,27) = 7.96, *p* < 0.001).

A lower average similarity was found when electrodes were virtually moved (IntraSessVER) inside the same session with respect to the original inter-session similarity (IntraSessORIG) without moving electrodes. Similarity values for IntraSessORIG and IntraSessVER (reported in Figure 16, Comp#2) were significantly different (F(1,9) = 432.68, *p* < 0.001). Also the VAF value (F(3,27) = 16.78, *p* < 0.001) and the interaction between type of comparison and VAF (F(3,27) = 36.79, *p* < 0.001 after sphericity correction) had a significant effect.

A similar analysis was applied to synergies from several sessions and different electrode positions in order to see if, by repositioning electrodes, it was possible to achieve higher similarity between two sessions. Similarity between two sessions with the original electrode positions (InterSessORIG) was compared (Figure 16, Comp#3) with similarity between the same two sessions while optimizing electrode positions in two sessions (InterSessVER). The difference was statistically significant (F(1,9) = 95.12, *p* < 0.001). While VAF also had a significant effect (F(3,27) = 4.89, *p* < 0.05 after sphericity correction), interaction was not significant (F(3,27) = 1.07, *p* = 0.36). This result suggests that it is possible that the lower similarity (Figure 16 Comp#1) that exists between inter-session (InterSessORIG) when compared with intra-session (IntraSessORIG) can be due to electrodes being slightly repositioned between sessions.

Even when optimally matching synergies between two sessions with virtual electrode repositioning (InterSessVER), it was not possible to achieve the similarity obtained in synergies computed from the same session after virtual electrode repositioning (IntraSessVER; Figure 16, Comp#4; F(1,9) = 62.76, *p* < 0.001). Interaction had significant influence (F(3,27) = 6.55, *p* < 0.05 after sphericity correction), but VAF did not (F(3,27) = 0.91, *p* = 0.41).

The last analysis proposed was to see how synergies from the same movements generalize between people (InterSUBJ). A comparison between the average subject-specific inter-session similarity (i.e., the similarity between synergies extracted for each person in different sessions, InterSessORIG) and the inter-subject similarity (Figure 16, Comp#5) revealed that there was no significant difference (F(1,9) = 0.46, *p* = 0.51). VAF influenced the similarity significantly (F(3,27) = 8.37, *p* < 0.001), but there was no interaction between VAF and the type of analysis (F(3,27) = 0.23, *p* = 0.055).

## 4. Discussion

An overview of previous studies revealed limited knowledge considering both inter-session and intra-session stability of synergies in healthy subjects. The novel contribution of this article is to clarify this difference using an appropriate dataset for both analyses. Intra-session stability of synergies is significantly higher than inter-session. There is a continuously decreasing trend of VAF CoV as the number of synergies increases, which stabilizes for a high number of synergies. A decreasing trend was observed as well by Taborri and colleagues [24] who studied intra-subject (intra-session) and inter-subject similarity during gait in healthy subjects. In another study [24], values of CoV for situations analogous to our intra-session comparison were maximally 1.7%, which is similar to values we detected for an equal number of synergies (3, 4, and 5) in our results.

A significant difference between inter- and intra-session and a similar decreasing trend was detected also when considering stability of NoS that are needed to explain at least 80, 85, 90, and 95% of variance of original EMG data. Variation of VAF was higher for smaller NoS, and variation of NoS was higher for smaller VAF. This result suggests that, choosing a higher VAF as criteria for selection of the number of synergies, the number of synergies is expected to be relatively stable in both experimental conditions. Instead, increasing the VAF value and accepting a “high” decomposition order reduces the potential of the muscle synergy approach. Thus, according to our results, using the VAF threshold as a method to determine the number of synergies across several sessions (possibly for inter-session comparison) might be a trade-off between stability of synergies and parsimonious number of modules.

The stability of VAF and NoS were highly informative but still could not provide details about the spatial synergy composition and the stability needed for multi-session assessments. Thus, the spatial synergy composition through sessions was compared using cosine similarity and matching.

The synergy similarity between sessions had a broad range from 0.7 to 0.85 (cosine similarity) depending on the subject and VAF. Possible explanations for differences between subjects are that some subjects had higher movement repeatability because of redundancy (or abundancy) of the musculoskeletal system that allows to perform a movement using different muscle recruitment options as suggested by Latash and colleagues [50] or because electrodes were not put exactly on the same position between sessions [20]. In order to understand and clarify this further, intra-session similarity analysis was performed.

We found that intra-session synergy similarity was higher than inter-session similarity. There are several possible reasons for finding significantly higher intra-session values compared to inter-session. First, the repeatability of EMG patterns can be higher inside the same session than between sessions. Since the subjects were instructed to perform exactly the same tasks during each session, this might happen because of natural variability of subjects or due to daily conditions, fatigue, or other factors related to each session. A second possible source of variability is slight electrode misplacement between sessions. Since even in the most accurate experimentation both factors can be found, it is challenging to control all the variables that may induce variability.

When synergies from the same session but different combinations of movement repetitions were used and compared (assessing intra-session variability), average similarity (for all sessions and participants) was around 0.962 ± 0.015. This value can be considered as the maximal similarity that we can expect between synergies from the same movement repeated several times. This is a very high similarity and such results have been reported previously [24,31] but using different similarity measures.

In the existing literature, studies analyzing inter-session or intra-session similarities were not performed on the same data and same muscles of interest. In all studies, it is claimed that good repeatability is found, both at the intra-session and inter-session levels. Kristiansen and colleagues [20] found very high repeatability of time activations of synergies between two sessions and smaller (even if still quite high) similarity of the spatial composition of synergies. Here, only two sessions were compared, whereas comparing more sessions as in our case can possibly lower the similarity.

Since the assessment of variability in stability and similarity of synergies comparing intra-session and inter-session conditions revealed significant differences, we also provided a simulation of displacement of the proximal bracelet of electrodes to question whether repositioning might influence these features of muscle synergies.

Comparing intra-session synergy similarity without electrode repositioning (IntraSessORIG) with electrode repositioning (IntraSessVER) allowed to detect a significant drop in average similarities when electrodes are repositioned (Comp#2). This leads to the conclusion that, if electrodes are displaced, we cannot expect that synergies can reach the similarity limit of 0.95 (average for all 10 subjects).

Inter-session synergy similarity significantly increased with virtual electrode repositioning compared to inter-session similarity with the original positions of the electrodes (Comp#3). We found that similarity of synergies between two sessions can be increased if electrodes of one session were virtually repositioned with the goal to achieve higher similarities. This opens a possibility that the lower inter-session synergy similarity found when compared with intra-session (Comp#1) similarity was due to the fact that electrodes were not positioned in exactly the same way for all sessions. This is further clarified in Comp#4. Observing that the highest scores in InterSessVER were smaller than the highest scores in IntraSessVER, we conclude that electrode repositioning cannot account for all the differences that exist between sessions. Possibly, residual differences can be explained by variability induced by changing conditions between sessions (e.g., fatigue of subject, concentration to movement performance, etc.) or because even more accurate simulations for electrode repositioning need to be used. For instance, while the simulated data accounted for linear, constant differences among the position of the electrodes, in reality, the variability of the positioning of the electrodes is most likely nonlinear and nonconstant also due to muscle activity and physiology.

We consider this result to be of interest in muscle synergy analysis. We found that, in applications that involve distal upper-limb coordination, part of the inter-session variability can be attributed to effects that are probably not related to real physiological variability in motor coordination. While this effect can be relevant in this application that involves fine control compared to others targeting other body segments, the inter-session protocol-related variability should induce researchers to interpret results collected at different time stages with particular care. Only after considering the difference in synergies between sessions due to such protocol-related reasons, one can focus on quantifying other changes of interest, such as recovery in case of patients.

Finally, after showing that the intra-session similarity is higher than inter-session similarity, we addressed whether synergy similarity can be distinguished when extracting synergies from the same person but in different sessions (inter-session) or extracting synergies from different people across sessions (inter-subject). We found the unexpected and very interesting result that there was no significant difference between the similarity of synergies from different people and the similarity of synergies from the same person, extracted in different sessions (Comp#5). This result can be interpreted under two perspectives: on one side, it confirms that synergies are meaningful and similar across subjects; on the other hand, while these results cannot be generalized to other scenarios and should be considered as specific of our application, they suggest caution for multi-session assessments and longitudinal analysis.

These results also suggest that, across sessions, the subjects’ synergies may have a comparable variability with respect to other people [24,25,50]. This result is not new and was found in this domain of application in recent studies at both the kinematic and EMG levels [51,52,53]. This effect may have implications in prosthesis and robot control based on the muscle synergy paradigms [54]. It is probably possible to find invariants shared across subjects, even if it can be with a broad tolerance. Implementing subject-specific assistance can be complex if such variability is detected at different times.

All the mentioned results suggest that the synergy-based evaluation of a person within the same session should consider the natural variability that underlies human motor control, as previously shown by other authors [5]. Even more importantly, several factors may affect the inter-session stability of synergies, and this point might have impact on longitudinal studies where synergies can be used for evaluation of neurological diseases at different time stages. The sources of variability may be endogenous (related to the subject’s daily variability, which is the one the experimenter is willing to capture; it might be related to the effects of a rehabilitation therapy) and exogenous (which is related to different electrode positioning that the experimenter wants to avoid since it biases the assessments). Electrode type and the person performing the experiment can also likely influence the evaluation. None of these factors should be a priori neglected, especially in inter-session assessments. Caution has to be taken in the interpretation of our results, since analysis was performed only on 10 healthy subjects. Moreover, in the simulated dataset, only 8 EMG channels were used for which the position is, first, variable between subjects due to anatomy and, second, specific for the prosthesis control applications, thus related to forearm. Lastly, it is important to note that artificially calculated EMG data for simulation of the movements of electrodes does not include any physiological information and we do not claim that this exact data would be recorded if the electrodes were really on these positions. This is an interesting possible (and recommended) next step of research. Finally, these results are related to NNMF and might differ for other algorithms and models. Therefore, while the introduced concept may have impact for the field of muscle synergies and rehabilitation, the presented results generalize only for the forearm and hand applications and may find applications including prosthesis [55] and movement intention detection [56]. This work will be the starting point for a more detailed analysis of hand grasps based both on muscle and kinematic patterns, as done on previous pioneering work [57], where it was demonstrated that a more compact representation and a higher decoding capacity of grasping tasks is found when movements are expressed in the muscle kinematics domain rather than kinematic alone.

## 5. Conclusions

In this paper, we analyzed robustness, stability, and similarity of the extracted muscle synergies under the effect of factors that may induce variability, including inter-session measurements and natural intra-session variability. These analyses can help shed light on the applicability and challenges of applying muscle synergies in clinical environments as well as to more deeply understand their functioning.

The main finding was that spatial similarity is significantly higher in intra-session conditions (synergies extracted from the same data acquisition) rather than in inter-session conditions (synergies extracted from the different data acquisitions). We also found that virtual electrode repositioning can significantly decrease the similarity of intra-session synergies, while it can also increase the similarity of synergies extracted in different sessions. Nevertheless, comparing the highest similarities achieved with virtual electrode repositioning for intra- and inter-sessions reveals that the electrode position does not fully explain the difference in similarity of inter-session compared to intra-session conditions. Future studies could explain if this is due to the strong simplification of the adopted modelling approach or to other experimental factors. Furthermore, the assessment of how well synergies generalize between healthy subjects while performing the same set of movements revealed no significant difference between the similarity of synergies from different subjects and the similarity of synergies from different sessions of the same subject. This point can have an impact on longitudinal studies where synergies can be used for the evaluation of neurological diseases at different time stages and for the assessment of treatment effects: if the changes we want to assess with the muscle synergy approach are smaller than this variability, then we will probably not be able to detect them. Lastly, the results are relative to the specific exercises and EMG channels recorded in the NinaPro hand grasp database 6 and cannot be extended to other movement tasks, electrode positions, and body segments. Nevertheless, they create a new understanding of the supporting evidence that muscle synergy analysis based on nonnegative matrix factorization is clinically and experimentally applicable with the limitations discussed in the paper. Further applications of our analysis need to be considered in longitudinal trials and clinical applications.
